# Acceptability of data linkage to identify women at risk of postnatal complication for the development of digital risk prediction tools and interventions to better optimise postnatal care, a qualitative descriptive study design

**DOI:** 10.1186/s12916-024-03489-7

**Published:** 2024-07-02

**Authors:** Siobhán O’Connor, George Tilston, Olivia Jones, Anita Sharma, Laura Ormesher, Bradley Quinn, Anthony Wilson, Jenny Myers, Niels Peek, Victoria Palin

**Affiliations:** 1 Division of Nursing, Midwifery and Social Work, School of Health Sciences, The University of Manchester, Manchester, M13 9PL UK; 2 Division of Informatics, Imaging and Data Science, School of Health Sciences, Faculty of Biology, Medicine, and Health, The University of Manchester, Manchester, M13 9PL UK; 3 Maternal and Fetal Health Research Centre, Division of Developmental Biology and Medicine, The University of Manchester, Manchester, M13 9WL UK; 4 NHS England, Manchester, UK; 5 Health Innovation Manchester, Manchester, M13 9NQ UK; 6 Clinical Data Science Unit, Manchester University NHS Foundation Trust, Manchester, M13 9WL UK; 7 The Healthcare Improvement Studies Institute (THIS Institute), Department of Public Health and Primary Care, University of Cambridge, Cambridge, UK

**Keywords:** Postnatal health, Digital health tools, Risk management, Maternal health, Cardiovascular disease, Risk prediction

## Abstract

**Background:**

Pregnancy acts as a cardiovascular stress test. Although many complications resolve following birth, women with hypertensive disorder of pregnancy have an increased risk of developing cardiovascular disease (CVD) long-term. Monitoring postnatal health can reduce this risk but requires better methods to identity high-risk women for timely interventions.

**Methods:**

Employing a qualitative descriptive study design, focus groups and/or interviews were conducted, separately engaging public contributors and clinical professionals. Diverse participants were recruited through social media convenience sampling. Semi-structured, facilitator-led discussions explored perspectives of current postnatal assessment and attitudes towards linking patient electronic healthcare data to develop digital tools for identifying postpartum women at risk of CVD. Participant perspectives were gathered using post-it notes or a facilitator scribe and analysed thematically.

**Results:**

From 27 public and seven clinical contributors, five themes regarding postnatal check expectations versus reality were developed, including ‘limited resources’, ‘low maternal health priority’, ‘lack of knowledge’, ‘ineffective systems’ and ‘new mum syndrome’. Despite some concerns, all supported data linkage to identify women postnatally, targeting intervention to those at greater risk of CVD. Participants outlined potential benefits of digitalisation and risk prediction, highlighting design and communication needs for diverse communities.

**Conclusions:**

Current health system constraints in England contribute to suboptimal postnatal care. Integrating data linkage and improving education on data and digital tools for maternal healthcare shows promise for enhanced monitoring and improved future health. Recognised for streamlining processes and risk prediction, digital tools may enable more person-centred care plans, addressing the gaps in current postnatal care practice.

**Supplementary Information:**

The online version contains supplementary material available at 10.1186/s12916-024-03489-7.

## Background

Cardiovascular disease (CVD) is a collective name for diseases of the heart and blood vessels such as coronary heart disease, cerebrovascular disease, peripheral vascular disease and heart failure. CVD is prevalent in developed countries and the leading cause of mortality worldwide [1], resulting in 17.9 million deaths each year [2, 3]. It is well recognised that there are sex-specific differences in when individuals will present with CVD [4, 5]. For women of childbearing age, pregnancy acts as a stress test on the body, where most organ systems are required to work harder to support the needs of the developing foetus [6]. As a result, some women develop complications during their pregnancy, such as hypertensive disorders of pregnancy, gestational diabetes, often associated with preterm, small or large for gestational age infants, as well as late pregnancy loss [7]. The stress of pregnancy on the body can also reveal underlying cardiometabolic disease risk, and whilst pregnancy complications (e.g. hypertensive disorders of pregnancy, gestational diabetes) usually resolve after birth, there is a growing body of evidence to suggest that women who experienced an adverse outcome of pregnancy have a greater risk of developing CVD in the next 10 years [7–9]. For example, hypertensive disorders of pregnancy leave mothers with a two- to four- fold increased risk of developing ischemic heart disease, stroke and heart failure postnatally [10–14], which increases with reoccurring events in subsequent pregnancies [15]. This clearly emphasises the importance of monitoring a woman’s health in the postnatal period, as well as a robust method to better identify women who are at increased risk of developing cardiometabolic disease following pregnancy such that women receive the provision of care required to improve long-term health.

During the postpartum period both mother and baby receive frequent assessments to ensure they do not experience short-term complications. In England, the National Institute for Health and Care Excellence recommends assessments are carried out in three stages. First, an assessment of mother and baby is conducted by a midwife within 24 h (a maximum of 36 h) after transfer of care from the place of birth (or home birth) and again at day five and 10; second, a health visitor assessment occurs approximately seven to 14 days after transfer of care from the midwifery team; and third, a postnatal assessment with a general practitioner (GP) six to eight weeks after the birth [16]. The timings of these checks are to identify immediate complications for mother and baby and to assess maternal physical and mental health. Although 75% of premature CVD is preventable through early risk factor modification [3], postnatal signs (or warning flags) are often neglected or mistaken for pregnancy associated adaptations [17]. In addition, postnatal review of blood pressure is often poorly monitored and inadequately treated.

Patient electronic health records (EHRs) and digital interventions to optimise care have become a large part of health research and clinical practice. EHRs are often used by clinical teams in hospital to record maternal and neonatal health events, support clinical decision-making and care delivery [18–20]. Beyond direct care, digital patient and administrative datasets can support secondary use, such as service evaluation and improvement [21, 22]. For instance the UK surveillance data is used for the annual MBRACE report, highlighting inequalities in maternal and neonatal morbidity and mortality by race [23]. Similarly, the Health Improvement Network, which holds over 16 million registered patients from 730 GP practices across UK, was used to examine women’s uptake of postnatal consultants after childbirth [24]. They found 20% did not have a consultation postnatally, estimating over 350,000 women per year in the UK may miss this important health check, highlighting the need for improved processes to ensure adequate timing of postpartum review. EHRs are also used for health research to improve outcomes for women and children. For example, the Fetal Medicine Foundation also utilised routinely collected EHRs to develop an algorithm to screen for risk of developing preeclampsia 11–13 weeks of gestation [25, 26]; this tool was shown to be more effective at identifying and reducing the risk of pre-eclampsia than standard care [27, 28]. In addition, machine learning algorithms applied to EHR data from primary care (> 18 million patients) were able to predict postpartum depression among women, a condition affecting mother-baby bonding, infant development and accounting for 20% of postpartum deaths [29]. This emphasise the potential advantages of incorporating such algorithms in the workflow of postpartum screening to identify women at risk of multiple complications in a timely manner.

Given the sensitivity of health records, data protection, security and patient trust are of utmost importance when conducting research studies and developing digital interventions to improve care. Secure data environments (SDE) now have the capabilities to link data from different services allowing clinical teams to quickly access patient records to support direct care [30]. Following the correct governance, infrastructures that allow secondary use of data for research have been established. These infrastructures may be utilised to securely link, analyse and develop novel predictive tools to identify mothers in the postpartum period with a greater risk of complication, who therefore require early intervention. Identifying those at highest risk of CVD and ensuring they receive appropriate treatment to improve overall health may help prevent premature death [31–33].

To develop such interventions, guidance is required to ensure secure data environments for research and the development of digital interventions for maternity services are considered acceptable and appropriate by service users, members of the public and clinical professionals. This research aimed to form two diverse groups of (1) public representatives of existing or potential users of maternity services and (2) clinical representatives (i.e. GPs and midwives). Within these groups, the care of women postnatally will be explored, with a specific focus on the acceptability and trustworthiness of data linkage to promote better digital health solutions for the improved identification and management of postnatal cardiometabolic disease and the risk reduction of future CVD events. Whilst data linked across health and care settings is widely seen as an enabler for the use of analytics for decision-making and improved healthcare delivery [34], there is a lack of knowledge for understanding the potential of data linkage and the implementation of digital tools for improving management of women postnatally and across maternity services in general. To our knowledge, this is the first study to explore the views of clinical professionals in addition to public representatives for the use of linked maternity data and risk management to improve healthcare delivery postnatally.

## Methods

### Design

An exploratory, descriptive study design was employed, and multiple focus group sessions or semi-structured interviews were conducted to understand the perspectives of both public and clinical stakeholders towards current postnatal care within general practice and the use of patient data to build a novel digital predictive tool that addresses the long-term risks of CVD in women post pregnancy.

### Sampling, recruitment and ethics

University Research Ethics Committee approval was obtained before recruitment (project reference: 2023–15928-27077). Convenience sampling targeted participants residing within or clinical professionals working within the Greater Manchester region of the UK via social media (X and Facebook) between 1^st^ March and 30^th^ June 2023. The recruitment advertisement and participant information sheet are available in the Supplement. All participants were invited to take part in face-to-face or virtual sessions, with public and clinical sessions conducted separately. Public contributors provided informed consent, whilst clinical stakeholders, recruited in line with their professional roles, implicitly consented through voluntary participation.

### Data collection

A total of seven facilitator-led (VP) sessions were conducted (Table 1) using a series of Microsoft PowerPoint presentations and activities, purposefully designed to collect participants’ perspectives in the style of a focus group with roundtable discussion to generate solutions to- and design of- a digital predictive tool. All data were collected with the use of a co-facilitator scribe (SO) and participants providing written comments on post-it notes.

**Table 1:** Summary of the session compositions

Session number	Form	Duration (min)	Public or clinical contributor	Number of attendees	Roles
1	In person	180	Public	5	Public
2	In person	180	Public	6	Public
3	Online	60	Clinical	1	GP
4	Online	60	Clinical	2	1 × hospital-based midwife 1 × community-based midwife
5	Online	60	Clinical	3	2 × GPs 1 × health psychologist
6	In person	150	Public	16	Public
7	Online	60	Clinical	1	GP

The three public sessions lasted approximately 150–180 min each and included a total of 27 participants. The following topics were explored:

- Maternal views of postnatal checks in general practice with regard to their awareness and expectations of these checks and the reality they experienced;
- Acceptability of using de-identified versions of maternal EHR within an SDE to develop mathematical algorithms that are able to predict individual risk for developing postnatal disease (using CVD as an example);
- Perspectives on developing digital interventions to link maternal hospital records with primary care physicians to automatically notify them that a woman had recently delivered and required medical review, including a predicted risk/score of developing CVD;
- Understanding of individual risk and preferences for timing and specifics of risk communication with healthcare professionals as well as patient facing interventions.

Four clinical sessions were conducted, taking place virtually over Zoom. A total of seven clinical participants attended with sessions lasting approximately 60 min each. Two sessions had only one clinical participant each and therefore a semi-structured interview style was adopted for these sessions. The following topics which were drawn from the scientific literature were explored:

- Current clinical practice in postnatal maternal health checks including notification processes, appointment scheduling, timing of checks, priority setting during consultations and current strengths and limitations in the process;
- Communication of long-term health risks to mothers postnatally;
- Acceptability of digital and/or other interventions that could streamline postnatal maternal health checks;
- Perspectives on potential interventions that would aid clinicians’ ability to monitor mothers and improve the delivery of maternal health services.

### Data analysis

The data from all public and clinical sessions were transcribed (SO) to Microsoft Excel. The data were analysed inductively using a thematic approach (SO) following the six phases outlined by Braun and Clarke [35]. This began through familiarisation with the data by reading and re-reading the field notes and recording preliminary ideas. Next, a set of initial codes were generated from relevant features in the dataset which were grouped logically together. Subsequently, general themes were formed in relation to the coded extracts of data to form an initial thematic map of the analysis. Each theme was then cross-checked against its coded extracts and subthemes created where necessary, before checking themes across the other themes and coded extracts to refine the thematic map and ensure data saturation was reached. Finally, a formal description and name for each theme was developed to help build a rich narrative of participant views and participant quotes were selected to support the themes. NVivo QSR 10.0 software was used throughout the qualitative analysis process.

### Rigour and reflexivity

Several techniques were used to enhance the study’s trustworthiness (i.e. credibility, dependability, confirmability and transferability) [36, 37]. Credibility was enhanced by employing robust data collection and analysis methods, with an informal debriefing session with a peer utilised to explore and minimise researcher bias. Dependability was improved using a coding clinic between the lead researchers (SO and VP) to check samples of data that were analysed. Transferability to other contexts was enriched through thick descriptions from participants on the acceptability of using patient data to build predictive tools to identify and communicate long-term risks to maternal health. Confirmability was possible due to the triangulation of data from a range of sources (i.e. public and clinical participants at the workshops) all of which helped improve the rigour of the qualitative findings. Furthermore, an international best practice guideline for reporting qualitative research, the COnsolidated criteria for REporting Qualitative research [38], was used to report the study’s results (see Supplement).

## Results

A total of 27 public contributors aged 25–55 years, and seven active clinical stakeholders were recruited. All participants attended and completed the sessions. For public contribution 96% were female and 86% for clinical stakeholders, who were a mixture of GPs, midwives and a clinical psychologist. For public contributors, 67% were of South Asian origin, 4% Black, with the remaining of White or mixed background. For clinical contributors, 71% were White, with the remaining mixed.

### Expectations versus the reality of postnatal checks

Five main themes on participant expectations of postnatal checks versus the reality were developed. These were (1) limited clinical resources with a focus on financial priorities, (2) low priority towards maternal health, (3) lack of knowledge, (4) ineffective systems and processes and (5) new mum syndrome. Each theme and associated subthemes are discussed below with supporting participant quotes shown in Table 2.

**Table 2:** Expectations versus the reality of postnatal checks

**Theme 1: Limited resources (e.g. staff, time) focused on financial priorities**
‘Postnatal is short staffed or can’t be bothered syndrome, not prepared, not up to date but just focusing on where the money is’ [WS6, GP, female] ‘One interpreter required per week for patients, in other GP practices about 40/50% patients require an interpreter e.g., repeat prescription takes 10 min versus 1–2 min with no translator’ [WS4, GP, male] ‘You can do the post-natal check, but 10 min is too short, and you need 20–30 min, and you can go through a detailed assessment and provide as assessment’ [WS6, GP, female]
**Theme 2: Lax approach to maternal health**
‘No opportunities to share as GP said “You look well”’ [WS2, public, female] ‘Severe pain from hernia, ring GP (it’ll just go down). Complained to a health visitor who arranged a scan to diagnosis hernia’ [WS3, public, female] ‘As you have more children, the assumption is you know everything, and you can do everything, when you are overwhelmed and down.’ [WS3, public, female]
**Theme 3: Lack of knowledge**
‘GP receptionist didn’t know about 6-week check when I rang’ [WS2, public, female] ‘No questions about feeding, sleeping, mental health’ [WS2, public, female] ‘First time mums are isolated, don’t know what questions to ask’ [WS3, public, female]
**Theme 4: Ineffective systems and processes**
‘We rely on mum and the discharge summary—you would have read the document beforehand but if it wasn’t clear on the discharge summary and the mum doesn’t bring it up and you have to go through each one to coax the information’ [WS4, male, GP] ‘EMIS has its own stock template which is horrible—all-encompassing has everything beyond the guidelines, text-boxes clunky’ [WS6, GP, female] ‘I don’t think we’re good at things after that initial period e.g., hypertension and it’s coded in the notes as a problem but in 12 months’ time I don’t know how good we are picking that up and she needs her BP checked’ [WS7, GP, female]
**Theme 5: New mum syndrome**
‘When you’re in pain and have poor mental health, you don’t have the headspace to make appointments and know what to ask. Mental load’ [WS2, public, female] ‘You’re too tired to chase up or go to another doctor’ [WS2, public, female] ‘Need 6-month check-up for mum, babies crying so by 3–6 months you know yourself how you are feeling, up to a year’ [WS3, public, female]

*Abbreviations*: *BP* blood pressure, *EMIS* Egton Medical Information System, *GP* general practitioner

#### Limited clinical resources with a focus on financial priorities

Several constraints with the six-to-eight-week postnatal check were voiced by both public clinical contributors which made them open to a digital risk prediction tool that could support maternal health. The first issue centred on the limited resources that were available in primary care to undertake detailed postnatal assessments such as a lack of experienced clinical and administrative staff, and short 10-min appointments which reduced the amount of time GPs had to build a rapport and discuss health issues in detail with women. Physicians in more deprived areas experienced additional language barriers to overcome which took up time during the consultation. In some cases, smaller GP practices had to prioritise high-risk women from their list of patients due to a lack of staff and were not able to accommodate everyone for a six-week check despite a desire to do so. Clinical stakeholders felt these problems may stem from the Quality and Outcomes Framework (QOF), a government reward programme which allocates money to GPs in England for delivering specific health services. As many GPs have a high workload, some choose to prioritise clinical work that is linked to the performance of their GP practice and financial incentives, over tasks such as postnatal checks for which there is no monetary reward.

#### Low priority towards maternal health

Another issue that was discussed were that some GPs did not seem to prioritise maternal health, with certain women describing the postnatal check as a ‘tick-box’ exercise that was not comprehensive (covering physical, mental and social health) or personalised. Others felt GPs could be dismissive of genuine problems or assumed low-risk women or those who already had children were not experiencing any issues. Both contributor groups felt postnatal GP-patient interactions were focused more on newborn health.

#### Lack of knowledge

The lack of knowledge of postnatal checks among child-bearing women, administrators and family doctors in primary care was also highlighted as a difficulty. Many public contributors acknowledged having no awareness of the six-week check or highlighted that they did not know what questions to ask their family doctor during the short assessment, particularly those who were not native to England. In some cases, this problem was compounded by administrative staff who were less familiar with the postnatal check, making it difficult for high-risk women to be prioritised for appointments. Several public contributors also raised concerns about GPs’ limited understanding of short- and long-term complications that could occur as well as a lack of physical examinations being conducted postnatally. In addition, they felt there was a lack of cultural awareness and other social issues among GPs that some women had to deal with in their local communities which affected their health. This included being examined by a male doctor and the need for more mental health support in communities where women were expected to function with minimal family and emotional support postnatally.

#### Ineffective systems and processes

Clinical stakeholders also mentioned several systems and processes in place in primary care which did not always support the delivery of postnatal checks. GP practices relied on hospital discharge summaries to identify women who needed a postnatal check, but these were not always complete, accurate or those that included short-term treatment plans were delivered too late or went astray. This could result in suboptimal or no follow-up for some women. In addition, discharge summaries were sometimes reviewed and electronically coded by administrative staff who might not be experienced enough to pick up all clinical problems, meaning GPs could miss important aspects of postnatal care. Electronic templates were also in place in some GP practices to facilitate the six-week check, but these could be difficult to use or depersonalise the assessment process, with some GPs ignoring them in favour of using their own clinical expertise.

#### New mum syndrome

Finally, several contributors referred to the challenges that women experience in the weeks and months after delivering a new-born baby, as they are expected to juggle caring and household responsibilities on little sleep, sometimes with limited support from partners or families, which can affect their mental health. This could mean women forget to contact their GP to book a six-week check, miss appointments or do not ask or answer pertinent questions during the assessment, due to physical and mental exhaustion or fear of being perceived as not coping.

### Data linkage to identify women at risk of postnatal complications

Four main themes were developed on participants perspectives of data linkage to identify women at risk of postnatal complications which were (1) supportive, (2) concerned, (3) indifferent and (4) entrepreneurial. Each theme and associated subthemes are discussed below with participant quotes to support each one (Table 3).

**Table 3:** Perspectives of data linkage to identify women at risk of postnatal complications

**Theme 1: Supportive**
Subtheme 1.1: Benefits mother and baby
‘How can things improve without research?’ [WS1, public, female] ‘Could be used in many years’ time for something good’ [WS2, public, female]
Subtheme 1.2: Benefits clinicians
‘Yes it would be useful because then the warning is coming and we would pick up those patients’ [WS6, clinician-GP, female] ‘I think to have that data you could pull down in terms of how many women have delivered etc., so women are not missed, that would be really good’ [WS7, clinician-GP, female]
**Theme 2: Concerned**
Subtheme 2.1: Inappropriate use of data
‘The fear of what individual’s information is being used for’ [WS1, public, female] ‘Don’t want to share data in case families and communities are harmed’ [WS2, public, female]
Subtheme 2.2: Risk of identification
‘Mistake in system (or human error), identifiable data is shared/leaked’ [WS1, public, female] ‘People are worried to be identified’ [WS1, public, female] ‘Afraid to give details of children—> thinking of their future (+ if there would be any way to later be identified)’ [WS1, public, female]
Subtheme 2.3: Data quality
‘How often is data updated and incorrect data deleted?’ [WS2, public, female] ‘Human error with data sharing and data entry’ [WS2, public, female]
Subtheme 2.4: Depersonalisation of healthcare services
‘Care isn’t personalised, you just fit the statistics and that’s your risk and you are treated this way’ [WS2, public, female] ‘Choice and consent is taken away now you fit into this box because research which will have a negative impact on women’ [WS2, public, female]
**Theme 3: Indifferent**
‘I couldn’t care less personally’ [WS1, public, female] ‘What can I do about long-term risks? Do I really need to know?’ [WS1, public, female]
**Theme 4: Entrepreneurial**
Subtheme 4.1: Education
‘Clinicians could revisit the specifics of data sharing with mums to help them see the benefits of sharing their data’ [WS2, public, female] ‘Ensure maximum awareness is raised across GM; to reassure people that their information is safe and unidentifiable’ [WS1, public, female]
Subtheme 4.2: Digital solution
‘Create an app where GP/Hospital can share information’ [WS1, public, female] ‘So I envisage having this system and having the codes for preeclampsia the senior admin team wouldn’t have to run their searches and do the Docman and they could access this system and use it to identify who to contact and the high risk could be quickly identified, they could do something with those risk thresholds to decide who needs to be seen urgently and booked in’ [WS5, clinician-GP, male]

*Abbreviations*: *Docman* Electronic Document Management, *GM* Greater Manchester, *GP* general practitioner

#### Supportive

Many of the public contributors expressed support for the secondary use of maternal data in health research and were positive about linking hospital maternity records electronically so they could be accessed by people undertaking research on women’s health postnatally. Some felt research was needed so that better outcomes for women and their babies could be achieved, and that linking digital health datasets could lead to long-term improvements in healthcare services. Similarly, several clinical stakeholders were supportive of linking health data for research purposes, as they could envisage how it would be useful for their own professional practice and improve the systems and processes currently in place in primary care for women’s health.

#### Concerned

Despite the supportive comments from contributors, some also expressed concerns around linking health data for research purposes. Firstly, several were worried that their health data might be used inappropriately by those who could access it. Some also shared specific anxieties around the inappropriate use of data, as it could negatively impact ethnic minority communities and families who were in vulnerable positions. Some public contributors also feared being identified in research through human or system errors if health data were not secure or if it were inadvertently or purposively leaked, as maternal health data can be sensitive in nature and the risk of identification could have negative implications for women. Poor data quality was another issue that was discussed, as health data is not always accurate and up-to-date, and mistakes can be made during data entry which could affect the quality of research on women’s health. Finally, the depersonalisation of services was also a concern for some public contributors as they were worried that statistics and other results from research could generalise women into specific categories that would dictate the care they received instead of it being tailored to their individual needs and values. No clinical stakeholder expressed concerns about linking maternal health data for research.

#### Indifferent

A handful of public contributors adopted a more relaxed view to linking and sharing data for research on women’s health as they did not hold a particular perspective for or against this approach.

#### Entrepreneurial

Both public and clinical contributors suggested ways to enhance the acceptability of linking data to identify women at risk of postnatal complications. Educating women and their families about the benefits of sharing health data was put forward as a way to tackle some of the misconceptions that exist on how and why health data is collected and used. Some felt clinicians would be best placed to inform women about the positive impact that using maternal health data for research could have, whilst others suggested a regional campaign or approach should be used to educate the public about the importance of linking health data for research. This could be tailored to specific communities to maximise inclusivity and reassure people that the process is secure.

### Digital risk prediction tool for maternal health

Three themes were developed around the acceptability of a digital risk prediction tool to identify women at risk of postnatal complications which were (1) potential benefits of digitalisation, (2) design considerations and (3) communication needs. Associated subthemes are discussed below with participant quotes to support each one (Table 4).

**Table 4:** Digital risk prediction tool to identify women at risk of postnatal complications

**Theme 1: Potential benefits of digitalisation**
Subtheme 1.1: Automatic notification and action plan
‘Some paper gets lost in the ether and gets missed and we don’t know why, electronic systems are better for not losing data’ [WS3, GP, male] ‘If someone did that calculation, rather than send a number, give info on what is needed for this woman, what action plan is needed around medication etc.’ [WS7, GP, female]
Subtheme 1.2: Women’s health
‘In general, post-natal delivery, care is very poor e.g., pre-eclampsia even if read coded they should be brought in for BP check, urine check etc.—there is no system at all’ [WS6, GP, female] ‘But this has to be provided – she’s 30 years old, she’s had a baby, she’s not well, a cardiovascular event as well, and if she has a cardio event in her 40’s and 50’s it affects the baby and whole household’ [WS6, GP, female]
Subtheme 1.3: Clinical continuity
‘It’s frustrating and if I got that, we would have that plan and we can follow up in 12 months’ time’ [WS7, GP, female]
**Theme 2: Design considerations**
Subtheme 2.1: Systems integration
‘If it could be on something we already go for things, but given the computer systems we have that could be challenging’ [WS7, GP, female] ‘System things and communication issues and the pressures that practices are under in deprived areas I’m not surprised about the lack of 6–8 week checks for mums’ [WS4, male, GP]
Subtheme 2.2: Speed of use
‘we have all these ideas and a perfect risk prediction model but your apt as a GP is only 10 min and it takes 2 min to document and it takes the lady 2 min to walk down and find my room so that doesn’t leave a lot of time, 6 min to punch in numbers in a risk prediction tool but perhaps a dashboard have it open alongside the postnatal checks but you need functionality that automates number entering and number crunching for it to float at the feasibility/testing stage’ [WS4, GP, male] ‘spend all your appointment explaining history with zero time to discuss actual problems’ [WS2, public, female]
Subtheme 2.3: Ease of use
‘If it’s not really easy, if it involves additional work for the clinician it won’t happen on the ground’ [WS7, GP, female] ‘How it goes to GP as a document—we get a list of 30–40 Docmans per day a list of discharge summaries, notifications of appointments with mental health, sift through them with varying degrees of concentration and if it’s very long I’ll click through it so having that info in a more readable and easy form that would be great’ [WS7, GP, female]
**Theme 3: Communication needs**
Subtheme 3.1: Multifaceted communications
‘Options and different formats for women e.g., conversation instead of stats’ [WS2, public, woman] ‘Infographics, imagery is powerful, colour palette (primary colours), podcasts from NHS, midwives could give out infographics’ [WS2, public, woman]
Subtheme 3.2: Positive framing
‘Flip good news around and risk so it’s not so negative’ [WS2, public, woman] ‘Visualisations might cause anxiety, should be combined with visualisations on solutions and lifestyle changes’ [WS1, public, woman]
Subtheme 3.3: Tailored communications
‘Risk prediction is personalised so I wouldn’t be saying to every woman that she’s at high risk if I had this tool’ [WS4, GP, male] ‘Clear information with tailored support so the info isn’t miscommunicated or misunderstood’ [WS2, public, woman]
Subtheme 3.4: Language barrier
‘It’s difficult to put a number or note or in predicting the future and some more educated people will be able to handle that information and other populations are less able to see it and they’ve got a new-born baby and you want to be positive’ [WS4, GP, male] ‘It can be difficult—if it’s BAME and she can’t understand and there is someone with her and then we talk to the interpreter first’ [WS6, GP, female]
Subtheme 3.5: Timing of communication
‘Another challenge—comparing it to the Q Risk—I ring them and they don’t want to know. The pharmacist rings them, its 50% and they don’t want to know’ [WS7, GP, female] ‘If we were targeting low risk women—we’ll probably tell them to lose weight and get exercise which they won’t want to do postnatally’ [WS7, GP, female]

*Abbreviations*: *BAME* Black, African and Middle Eastern, *BP* blood pressure, *Docman* Electronic Document Management, *GP* general practitioner, *NHS* National Health Service

#### Potential benefits of digitalisation

Both public contributors and clinical stakeholders reported several benefits that might be gained from developing a digital risk prediction tool making it more acceptable to them. One advantage of an electronic system that was expressed was that automatic notifications could be sent to GPs highlighting women at risk of postnatal complications and an action plan based on individual risk factors could accompany this, particularly if a tool could scan one’s medical history to inform all aspects related to risk. This could avoid paperwork going astray, improve the quality of patients’ information, speed up the coding of discharge referrals from hospital based maternity services to reduce the possibility that high-risk women may be overlooked by administrative staff, and support GPs to undertake a thorough postnatal assessment. Furthermore, some clinicians felt providing a single risk score was not enough and a digital tool such as a patient facing app should also recommend personal lifestyle, medication and other changes that women could make to tackle the long-term risk of cardiovascular and other diseases. This level of detail would not only improve women’s health but could also positively impact those of their children and family, given the caring responsibilities many women take on in their homes and communities. Continued clinical contact was another potential benefit of a digital tool put forward by participants, as some thought it would enable GPs to provide long-term follow-up more easily by identifying those who need regular review.

#### Design considerations

A number of design considerations were also mentioned in relation to creating and introducing a digital risk prediction tool in primary care which could affect its acceptability. Systems integration was one issue raised by several clinicians as they thought it was important that any new digital tool was integrated with current computer systems in GP practices as this would improve the likelihood of them using it and ensure postnatal checks on all mothers were completed. However, one GP noted that interoperability could be challenging given the diversity of technologies used in primary care. A busy workload was another challenge mentioned as appointments are often limited to 10–15 min per person, reducing the amount of time a GP has to use a digital risk prediction tool and discuss the results of this with women postnatally. The usability of a new digital tool was also highlighted as several GPs emphasised the importance of having something that was accessible and easy for them to use, with one suggesting the risk score could form part of the electronic discharge referral that they received when a new mother went home from hospital-based maternity services.

#### Communication needs

Communication was also seen as a key factor for both public contributors and clinical stakeholders when employing a digital tool to facilitate discussions around long-term postnatal risks and ensure these are managed appropriately. Multifaceted communications were discussed in numerous workshops with the public contributors recommending a range of approaches be used to converse with women from different backgrounds about long-term postnatal complications. For example, the use of infographics was suggested as a format that might suit some people, along with conversations instead of providing statistics on the risk of CVD to help women understand how risk could affect them. Several public contributors advised that information about risk should be framed in a positive way to prevent causing anxiety to new mothers and constructive feedback provided about what actions women can take to address any long-term risks.

Tailoring communications to individual women was also highlighted as important when discussing long-term risk, so that women who were high risk could be targeted and the information conveyed is clearly understood. This might involve personalising how risk information is shared with some suggesting using community groups or health visitors to reach specific women rather than it being done in a consultation with a family doctor. Another consideration when communicating risk prediction and its associated management was language barriers that sometimes arose. This could occur with women whose native language was not English or for those less well educated as they may struggle to understand complex information. Finally, there were varying views on when women should be informed of long-term health risks, with some public contributors suggesting this should be communicated one month, six months or 12 months postnatally which could be facilitated via a digital tool. In certain instances, some women did not want to hear about these risks immediately after birth and thought it would be more appropriate if they were discussed at a later stage.

## Discussion

### Principal findings

The reality of postnatal examinations in general practice at six-to-eight weeks differed significantly from woman’s expectations, with many public contributors describing them as a mere ‘tick-box’ exercise lacking comprehensiveness or personalisation. Clinical stakeholders highlighted ineffective processes and system constraints hindering detailed examinations. Concerns about data security and inappropriate use emerged when linking maternity data between care sites. Despite these concerns, all contributors supported better education of women and families about the benefits of sharing health data. Tailored educational campaigns to specific communities were suggested to maximise inclusivity. Contributors emphasised the challenges related to system integration, crucial design aspects and the need to customise designs to align with the requirements of individual communities. Both public and clinical contributors also recognised the potential benefit of digital risk prediction tools to help streamline and automate processes to identify those at greater risk earlier, whilst facilitating the development of individualised care plans and personalised lifestyle recommendations.

### Comparison with existing literature

Consistent with our findings, a recent survey of over 2500 birthing parents revealed that not all GP practices comply with the requirement to provide a postnatal check, and among those that do, awareness of the examinations national guidance may be lacking [39]. Clinical contributors described the current built-in tools within the practice’s computer management systems as excessive and cumbersome, and challenging to complete within a time-restricted consultation. Unsurprisingly, resulting in a ‘tick-box’ experience lacking comprehension and personalisation, with some examinations and conversations being overlooked [39, 40].

Despite clinicians’ desire to deliver all essential services, there is concern that the increasing burden on primary care has resulted in a shift in practice where consultations for medical conditions covered by the QOF are prioritised due to the government’s financial reward system for the performance management of general practitioners in England [41], leading to the neglect of patient needs and concerns [42]. Notably, there does not currently appear to be a QOF metric specifically addressing postnatal care for women and birthing parents, potentially diminishing its importance and fuelling inconsistencies in completion.

Pivotal aspects of the NHS Maternity Transformation Programme aim to standardise and digitalise women’s access to their electronic maternity record by 2023/2024 [43, 44]. The plan focuses on providing women with more ‘personalised care’ and ensuring ‘better equality in management and treatment for mother and baby’ [44]. Given the global burden of CVD and a recent increase in CVD-related mortality in developed countries [45], early identification of individuals at risk is crucial. Predictive clinical models are increasingly adopted into routine clinical care [46]. However, few predictive models exist for development of maternal postnatal disease. Current risk assessment often necessitates expensive and complex clinical examinations to assessing cardiac function, which cannot be conducted in community practice [47]. Future research should examine the development of quick and assessable tools for risk assessment and management for primary care clinicians.

Clinicians recognised the significant value of a digital tool capable of linking data from recent hospital examination and scanning a woman’s electronic health record to present past medical history quickly and informatively. This approach enhances the available information, enabling the creation of a personalised summary. This in turn will empower GPs to formulate optimised management plans with the woman during time-constrained appointments. Our clinical stakeholders had no concerns about linking data for research, suggesting they are more familiar with the process than the public and therefore may have better comprehension of the ethical considerations and data management systems in place to protect health data. Primary care digital knowledge support systems [48, 49] are poised to be particularly effective in managing postnatal care, given the individual variations in women’s experiences during pregnancy and labour, considering specific medical events that may influence both immediate and future postnatal treatment.

Web-based electronic dashboards have shown to improve delivery of care by supporting medication reviews, optimisation and the personalisation of health delivery in primary care [50, 51]. Patient-focused digital interventions have also shown promise for the optimisation of healthy dietary behaviours and decreased weight gain during pregnancy [52] as well as being effective in preventing postpartum depression [53]. However, the widespread uptake of such digital technologies can be challenging if there are limited resources on the ground and when delivery requires heavy investment from the end users [54, 55]. Whilst there is a willingness of clinicians to embrace complementary digital tools in primary, it is crucial that these tools complement face-to-face clinical encounters and serve as facilitators rather than burdens [56]. However, there is a notable lack of evidence either substantiating or discouraging the use of digital interventions in relation to improved outcomes. Especially concerning their impact on quality of care, service delivery, benefits or harms to patients, or their cost-effectiveness [57]. Future research is required to estimate the effectiveness of such interventions for postnatal health and establish robust methods to evaluate interventions, especially for long-term CVD risk prevention, such as the use of surrogate endpoints, or modelling short-term endpoints (based on newly collected data) to extrapolate and update estimated long-term outcomes. Encouragingly, a systematic review found that younger and female patients are more likely to interact with alternative digital modes of communication in the primary care setting [57], suggesting that women of childbearing age may be the optimal target population for digital intervention and improvement tools in primary care. One main conflict we observed, requiring further evidence synthesis, is to determine the right format and timing of patient-facing information about postnatal health risks for mothers [58]. Promisingly, a digital tool to better optimise data linkage between maternity care sites, notify general practitioners of mothers and babies requiring review, and personalise healthcare plans could significantly improve the delivery of postnatal care. This approach would allow healthcare providers to tailor care to individual patient’s needs and could be pivotal to instigating preventative interventions to reduce long-term CVD risk.

A recommendation for future implications could involve a revision to the QOF to incorporate a specific metric for maternal postnatal care. This adjustment would address the current gap in evaluating and incentivising postnatal care within the framework. More research would be required to understand the implications of the addition of postnatal care metrics but does present an opportunity to refine healthcare policies and practices, ultimately leading to improved maternal long-term health outcomes.

### Strengths and limitations

There were several strengths and limitations to the study. Robust methods of data collection and analysis were adopted, and a reasonable sample size was achieved enabling an in-depth exploration of the acceptability of linking secondary maternal health data to develop risk prediction tools to improve postnatal care. The study was conducted by an experienced research team from a variety of scientific disciplines (e.g. medicine, nursing, computer science, digital epidemiology and health information science and systems) who discussed and agreed the interpretation of data and the study’s findings. Whilst the public contributors were a diverse group, they self-selected to participate in the study, and only a small sample of clinicians were represented all of whom were female bar one. In addition, all participants were drawn from the north-west of England and policy makers, who could offer additional insights into postnatal care, were not included in the sample. This could mean the findings may contain some bias and should be interpreted with caution. Furthermore, more detailed sociodemographic characteristics such as education level were not collected which could affect how well some participants understood the concepts being discussed. However, the research team explained the rationale for the study in clear and simple language during short presentations to participants and provided opportunities for questions to be asked helping to mitigate any misunderstanding of complex terminology related to digital health.

## Conclusions

Current limitations in England’s health systems often result in postnatal examinations falling short of women’s expectations, lacking comprehensiveness and personalisation. Integrating health data across care sites, along with improved education on the use of data and digital tools to support maternal healthcare delivery, has the potential to enhance postnatal care. The design of these digital tools must address challenges such as system integration and inclusiveness. Whilst some concerns about data security, data quality and inappropriate use were expressed, public and clinical attitudes towards digital interventions in maternity care were overall positive. By recognising benefits for streamlining referral and assessment, as well as identifying and managing women at a higher risk of developing postnatal disease, such as CVD, digital risk tools have the potential to improve postnatal care. In this way, they could enable more person-centred care plans, ultimately contributing to improved future health outcomes.

### Supplementary Information

Additional file 1: Supplement 1 Social media ad for PPIE.

Additional file 2: Supplement 2 Social media ad for clinical staff.

Additional file 3: Supplement 3 PPIE information sheet.

Additional file 4: Supplement 4 COREQ checklist.

## Data Availability

The datasets generated and/or analysed during the current study are not publicly available to protect individual participant privacy and prevent this being compromised.

## Abbreviations

BAME: Black, African and Middle Eastern
BP: Blood Pressure
CVD: Cardiovascular disease
Docman: Electronic Document Management
EHR: Electronic Health Records
EMIS: Egton Medical Information System
GM: Greater Manchester
GP: General Practitioner
MBRRACE: Mothers and Babies: Reducing Risk through Audits and Confidential Enquiries
NHS: National Health Service
QOF: Quality and Outcomes Framework
SDE: Secure Data Environment
