# Supplementary material for: Acceptability of data linkage to identify women at risk of postnatal complication for the development of digital risk prediction tools and interventions to better optimise postnatal care, a qualitative descriptive study design
Source: BMC Med. 2024 Jul 2;22:276. doi: 10.1186/s12916-024-03489-7 (PMC11220952; doi:10.1186/s12916-024-03489-7)
Supplement: Supplementary file 1 — Additional file 1: Supplement 1 Social media ad for PPIE. [file 12916_2024_3489_MOESM1_ESM.pdf]

MANCHESTER 1824

The University of Manchester

Do you live in Greater Manchester?

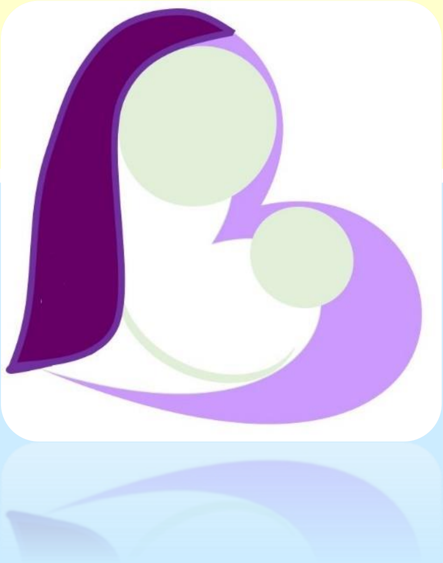

Are you aged 18-45 and passionate about maternity services?

PARTICIPANTS NEEDED

Members of the public are invited to attend a workshop that aims to inform and improve the way postnatal maternity care is delivered across Greater Manchester

<insert date and time of workshop>

Some women can develop a complication during their pregnancy.
Whilst most mothers recover, some complications may impact the future health of mums, putting them at a higher risk of developing a long-term health condition.

A workshop run by Manchester University will explore the acceptability of:

- using patient records to identify mothers in the postpartum period,
- using de-identified versions of these records to mathematically predict individual risk for developing postnatal disease,
- prioritising postnatal care based on this risk

For more information or to get involved, please email the research team

<insert contact email>

Please also see the University of Manchester's Privacy Notice for Research: https://documents.manchester.ac.uk/display.aspx?DocID=37095
