# Supplementary material for: Acceptability of data linkage to identify women at risk of postnatal complication for the development of digital risk prediction tools and interventions to better optimise postnatal care, a qualitative descriptive study design
Source: BMC Med. 2024 Jul 2;22:276. doi: 10.1186/s12916-024-03489-7 (PMC11220952; doi:10.1186/s12916-024-03489-7)
Supplement: Supplementary file 2 — Additional file 2: Supplement 2 Social media ad for clinical staff. [file 12916_2024_3489_MOESM2_ESM.pdf]

MANCHESTER 1824
The University of Manchester

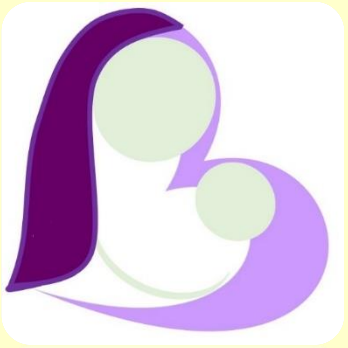

Are you a GP, obstetrician or midwife working in Greater Manchester?

PARTICIPANTS NEEDED

<insert date and time of workshop>

Clinical staff are invited to attend a face-to-face workshop that aims to improve maternity services across Greater Manchester

Attending a face-to-face workshop (2.5h) with other clinical professionals to express your opinions and help improve the continuity of care for mothers postnatally in GM.

Help develop a digital tool that can run predictive algorithms on linked patient data to identify women at risk of postnatal morbidity.

Is it acceptable to stratify care based on risk?
How best do we communicate this risk?

For more information and to take part please email the research team <insert contact email>

Please also see the University of Manchester's Privacy Notice for Research:
https://documents.manchester.ac.uk/display.aspx?DocID=37095
