# Supplementary material for: Acceptability of data linkage to identify women at risk of postnatal complication for the development of digital risk prediction tools and interventions to better optimise postnatal care, a qualitative descriptive study design
Source: BMC Med. 2024 Jul 2;22:276. doi: 10.1186/s12916-024-03489-7 (PMC11220952; doi:10.1186/s12916-024-03489-7)
Supplement: Supplementary file 3 — Additional file 3: Supplement 3 PPIE information sheet. [file 12916_2024_3489_MOESM3_ESM.pdf]

The University of Manchester

Faculty of Biology, Medicine, and Health's
Information Sheet for Public Contributors for Patient and Public Involvement and Engagement (PPIE) research

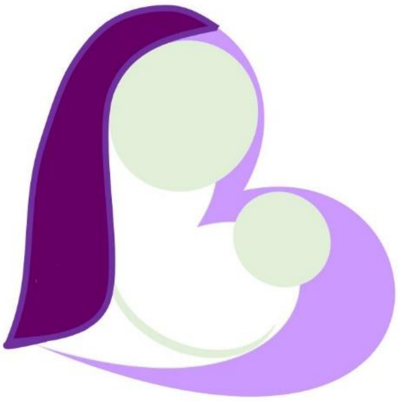

"Defining the acceptability of 'safe' data linkage to identify women at risk of postnatal complication in Greater Manchester"

Do you live in Greater Manchester? Are you ages 18 – 45 years?

You are being invited to be involved in a Patient and Public Involvement and Engagement (PPIE) workshop. This workshop aims to bring members of the public together to explore the acceptability of safely linking patient health records together to improve clinical practice in your region.

Some women can develop a complication during their pregnancy. Whilst most women go on to have a normal pregnancy and a full recovery, some complications women experience may impact their future health, putting them at a higher risk of developing a long-term health condition. For example, mothers that experience a high blood pressure during their pregnancy have a greater chance of developing cardiovascular related disease as they age. Research has shown that using linked patient health records and monitoring individuals closely at times of need can improve longevity.

This workshop aims to explore the opinions from members of the public to improve the way mothers are monitored and reviewed to identify those at risk of developing long-term health conditions after having a baby.

We are keen to involve a diverse population, including a range of different ages, ethnicities, and male and female participants. Before you decide whether you wish to take part, please take the time to carefully read through this information. It will help to explain why the research is being done and what it will involve. Please ask the research team as many questions as you wish. Please ask if anything is unclear or if you would like more information.

About the activity

Who will conduct the activity?

The workshop will be led by Dr Victoria Palin and Dr Siobhán O'Connor, lecturers from The University of Manchester. Dr Palin is a lecturer in Maternal and Fetal Health and Dr Siobhán O'Connor, a senior lecturer in nursing. Both researchers have experience looking at ways in which we can help to improve the health of mother and baby during and immediately after a pregnancy by analysing patient electronic health records and making recommendations to clinical care providers.

What is its purpose?

This study is seeking advice from members of the public to help in the development of digital tool that GPs can use to improve the way they monitor women after a pregnancy. By targeting this aftercare to women with a higher risk, we hope to reduce the number of women that go on to develop a long-term health condition. This workshop aims to gain public opinions to help understand:

- If it is okay to link patient records between primary (GP practices) and secondary (hospitals) care for mothers during and after a pregnancy
- Is it acceptable to use de-identified versions of these records to apply mathematical analyses to develop ways to identify women with greater risk of developing a long-term health condition.
- Is it okay to use the results from this analysis to help build digital tools for clinical care that notify doctors which women have a greater risk
- Should appointments be prioritised based on this risk.
- To ensure the digital tools communicate patient risk in a way that is easy to understand for patients without causing unnecessary stress.

My involvement

What would I be asked to do if I took part?

You would be asked to attend a workshop in person at The University of Manchester. The workshop will involve approximately 10 members of the public (including you) and last around 2.5 hours. There will be refreshments and facilities available.

The workshop will be led by Dr Palin and Dr O'Connor, who will guide the group through a series of topics for discussion. There will also be a few additional members of staff (from the research team) there to help deliver the workshop and to answer any question you may have throughout the session. The workshop will be delivered using short presentations, handouts, and group exercises. These will include writing ideas on post-it notes and flip charts.

During these sessions you are asked to contribute your opinions for each of the topics discussed. The workshop is a safe space to contribute to and voice your opinions. All information you and other volunteers contribute will be analysed after the workshop to bring out key themes. For this reason, the research team will be taking notes throughout the session and all additional materials created through each activity (papers, post-it notes etc.,) will be collected at the end of the session.
Any identifiable information (e.g., a name) will be removed from this material following the workshop before analysis. This ensures all contributions are anonymous. On occasion, some direct quotes may be used (as an example) to demonstrate some of the themes identified, however these will be anonymous, ensuring the quote does not include any identifiable information the participant may have shared.

Attending this workshop will allow you to contribute to the understanding of the research team. The results of this study will lead to the design of a digital tools that combines important information from public representatives (you) with the clinical need.

How long will the activity take and where will it take place?

Public volunteers will be recruited during February and the workshop will be held in March 2023. During the recruitment phase, each participant will read the study information pack and sign a consent form. You would then be asked to attend in person to one workshop at The University of Manchester. We are running multiple workshops between March and April 2023. We aim for the workshop to take 2.5 hours but will last no more than 3 hours. We will provide refreshments and there will be access to facilities.

Will I be paid for taking part?

Each member of the public will be compensated for their time, receiving £75 for attending and participating in one workshop. In addition, reimbursement of travel costs across Greater Manchester (up to £10) and childcare (when applicable) are available. On completion of the workshop, an expense form will be completed and processed. Funds will be paid directly to you via bank transfer.

What happens if I do not want to take part or if I change my mind?

It is up to you to decide whether to take part. You will be given at least 24 hours from receiving this information to consider if you wish to participate. You can inform us whether you want to take part or not via email. If you do decide to take part, you will be given this information sheet to keep and will be asked to sign a consent form. If you decide to take part, you are still free to withdraw at any time without giving a reason and without detriment to yourself. However, it will not be possible to remove your data from the project once it has been anonymized as we will not be able to identify your specific data. This does not affect your data protection rights. If you decide not to take part, you do not need to do anything further.

Data Protection and Confidentiality

What information will you collect about me?

To volunteer we will need to collect information that could identify you, called “personal identifiable information”. We will only ever collect information that is appropriate and necessary.
Specifically, we will need to collect:

- A record of your consent
- Your age and sex
- Your contact details, e.g., an email address or mobile phone number
- The council you reside in in Greater Manchester (e.g., Stockport)

Under what legal basis are you collecting this information?

Data protection law requires us to have a valid legal reason to process and use personal data about you, known as a “legal basis”. For PPIE activities, the specific reason is that it is “a public interest task” and “necessary for the pursuit of the legitimate interests of the University (i.e., PPIE)”.
We may also use your personal information in the following situation, which is likely to be rare: “Where we need to protect your vital interests (or someone else’s interests)”
If we require your consent for any additional uses of your personal information, including your image and more sensitive personal information (e.g., ‘Special Category Data’) we will obtain it.

What are my rights?

By law you have rights in relation to the personal information we hold about you. These include the right to:

- See the information/receive a copy;
- Correct inaccurate information;
- Have any information deleted;
- Limit or raise concerns to our processing of the information;
- Move your information ('portability')

The above would be granted unless it is legally prohibited and/or would put you or another person at serious risk of harm. To find out more about your different rights or the way we use your personal information to ensure we follow the law, please visit our PPIE Privacy Notice: http://documents.manchester.ac.uk/display.aspx?DocID=43063.

Will my involvement be confidential, and my personal identifiable information be protected?

Your personal information will be always kept confidential. The University as a Data Controller will remain responsible for keeping your information safe. We would not share information with other people or organisations about you or pass on your contact details without your permission. All staff members are trained in data protection, and your data will be looked after in the following way:

- All contributions provided by participants during the workshop (verbally or written) will be collected at the end of the session by the research lead.

- These contributions will be de-identified (anonymised) by the research team by removing any identifiable information (e.g., names, age, sex etc.) and electronically scanned.

- Scanned digital copies of the de-identified documents will be stored within The University of Manchester's Research Data Management Service (RDMS), which provides robust, managed, secure, replicated storage. Access to the data servers is restricted and only available to the research team. No data will be saved or stored or local or mobile drives.

- De-identified data will be retained until analysis is complete and summary findings are published in a peer-reviewed scientific journal, typically within 6-12 months of the data collection but may be retained for up to a maximum of 5 years.

- Hard copied of the data following de-identification will be destroyed once digital copies have been stored within the RDMS.

Potential disclosures:

- If during the workshop we have concerns about your safety or wellbeing we may need to contact someone outside of research team (e.g., a family member) on your behalf.
- Please note, we ask all participants to be professional and respectful to each other during the workshop's discussions. It is expected that all opinions or personal experiences shared during discussions will be kept confidential by all participants.
- Please also note that individuals from The University of Manchester or regulatory authorities may need to look at the data collected for this project to make sure it is being carried out as planned. This may involve looking at identifiable data. All individuals involved in auditing and monitoring the project will have a strict duty of confidentiality to you.

Will the outcomes of the activity be published?

The outcomes of this work will inform the development of a digital tool, with the aim that any concerns and/or recommendations identified by members of the public during the workshops will be incorporated into the design. In addition to this, a summary of the results will be published in research paper. This means the study's results will be published online through the medical journal's website. No data published will allow contributors to be identified in any way. Only summary information of the key findings/themes the emerge during the discussions will be published. All identifiable information will be removed. If you agree to take part in the study, you will be provided with research team contact details. You are welcome to contact any of the named persons if you wish to access the results of this study.

Contact Details

If you have any queries about the information provided or you are interested in taking part, then please contact

Name: **Dr Victoria Palin**
Email: **victoria.palin@manchester.ac.uk**

What if I have a complaint?

If by any means you wish to make a formal complaint, all participants can direct their complaint directly to the lead investigator (Dr Victoria Palin)

Name: **Dr Victoria Palin**
Email: **victoria.palin@manchester.ac.uk**

If you wish to make a formal complaint related to activity, or if you are not satisfied with the response you have gained in the first instance, then please contact:

The Social Responsibility and Public Engagement Team, Faculty of Biology, Medicine and Health, Simon Building, The University of Manchester, Oxford Road, Manchester, M13 9PL, by emailing: srbmh@manchester.ac.uk or by telephoning 0161 306 6797

If you wish to contact us about your data protection rights, please email dataprotection@manchester.ac.uk or write to The Information Governance Office, Christie Building, The University of Manchester, Oxford Road, M13 9PL at the University and we will guide you through the process of exercising your rights.

You also have a right to complain to the Information Commissioner's Office about complaints relating to your personal identifiable information https://ico.org.uk/make-a-complaint/ Telephone: 0303 123 1113
