# Supplementary material for: Acceptability of data linkage to identify women at risk of postnatal complication for the development of digital risk prediction tools and interventions to better optimise postnatal care, a qualitative descriptive study design
Source: BMC Med. 2024 Jul 2;22:276. doi: 10.1186/s12916-024-03489-7 (PMC11220952; doi:10.1186/s12916-024-03489-7)
Supplement: Supplementary file 4 — Additional file 4: Supplement 4 COREQ checklist. [file 12916_2024_3489_MOESM4_ESM.pdf]

Appendix X. Consolidated criteria for reporting qualitative studies (COREQ): 32-item checklist.

No	Item	Guide questions/description	Notes
**Domain 1: Research team and reflexivity**			
Personal Characteristics			
1	Interviewer/facilitator	Which author/s conducted the interview or focus group?	VP and SO
2	Credentials	What were the researcher's credentials? e.g., PhD, MD	VP and SO are both PhD qualified
3	Occupation	What was their occupation at the time of the study?	VP and SO were both health researchers, with SO also being a registered nurse
4	Gender	Was the researcher male or female?	Both VP and SO are female
5	Experience and training	What experience or training did the researcher have?	Both VP and SO are PhD qualified, with more than 5-10 years' experience conducting health research
Relationship with participants			
6	Relationship established	Was a relationship established prior to study commencement?	No relationship to participants
7	Participant knowledge of the interviewer	What did the participants know about the researcher? e.g., personal goals, reasons for doing the research	The rationale for the research study was explained to participants
8	Interviewer characteristics	What characteristics were reported about the interviewer/facilitator? e.g., Bias, assumptions, reasons, and interests in the research topic	None
**Domain 2: study design**			
Theoretical framework			
9	Methodological orientation and Theory	What methodological orientation was stated to underpin the study? e.g., grounded theory, discourse analysis, ethnography, phenomenology, content analysis	None
Participant selection			
10	Sampling	How were participants selected? e.g., purposive, convenience, consecutive, snowball	Convenience
11	Method of approach	How were participants approached? e.g., face-to-face, telephone, mail, email	Social media
12	Sample size	How many participants were in the study?	34 (27 public and 7 clinical participants)
13	Non-participation	How many people refused to participate or dropped out? Reasons?	There were no dropouts and 3 refused due to professional commitments.

Setting			
14	Setting of data collection	Where was the data collected? e.g., home, clinic, workplace	Public focus groups were conducted face-to-face. Focus groups and interviews with clinicians were conducted online using MS Teams/Zoom
15	Presence of non-participants	Was anyone else present besides the participants and researchers?	For one public focus group, two colleagues attended to present an overview of secure data environments and maternal cardiovascular health.
16	Description of sample	What are the important characteristics of the sample? e.g., demographic data, date	Age, gender, and ethnicity data were gathered. These are reported at the beginning of the Results section.
Data collection			
17	Interview guide	Were questions, prompts, guides provided by the authors? Was it pilot tested?	Yes - questions were provided and these were piloted tested amongst the research team
18	Repeat interviews	Were repeat interviews carried out? If yes, how many?	No
19	Audio/visual recording	Did the research use audio or visual recording to collect the data?	No – participant feedback was gathered using post-it notes
20	Field notes	Were field notes made during and/or after the interview or focus group?	Yes
21	Duration	What was the duration of the interviews or focus group?	Focus groups lasted 120-180 minutes; Interviews lasted approximately 60 minutes
22	Data saturation	Was data saturation discussed?	Yes
23	Transcripts returned	Were transcripts returned to participants for comment and/or correction?	No
**Domain 3: analysis and findings**			
Data analysis			
24	Number of data coders	How many data coders coded the data?	One with samples cross-checked by a second researcher
25	Description of the coding tree	Did authors provide a description of the coding tree?	No
26	Derivation of themes	Were themes identified in advance or derived from the data?	Derived from the data (inductive coding)
27	Software	What software, if applicable, was used to manage the data?	Yes – Microsoft Excel

28	Participant checking	Did participants provide feedback on the findings?	No
Reporting			
29	Quotations presented	Were participant quotations presented to illustrate the themes / findings? Was each quotation identified? e.g., participant number	Yes – please see the Results section of the paper
30	Data and findings consistent	Was there consistency between the data presented and the findings?	Yes – please see the Results section of the paper
31	Clarity of major themes	Were major themes clearly presented in the findings?	Yes – please see the Results section of the paper
32	Clarity of minor themes	Is there a description of diverse cases or discussion of minor themes?	Yes – please see the Results section of the paper
